# Developing Core/Shell Capsules Based on Hydroxypropyl Methylcellulose and Gelatin through Electrodynamic Atomization for Betalain Encapsulation

**DOI:** 10.3390/polym15020361

**Published:** 2023-01-10

**Authors:** Carol López de Dicastillo, Eliezer Velásquez, Adrián Rojas, Luan Garrido, María Carolina Moreno, Abel Guarda, María José Galotto

**Affiliations:** 1 Packaging Laboratory, Institute of Agrochemistry and Food Technology (IATA-CSIC), Av. Agustín Escardino 7, 46980 Paterna, Spain; 2 Packaging Innovation Center (LABEN), Department of Food Science and Technology, Technology Faculty, University of Santiago de Chile (USACH), Santiago 9170201, Chile; 3 CEDENNA (Center for the Development of Nanoscience and Nanotechnology), Santiago 9170124, Chile; 4 Department of Chemical and Bioprocess Engineering, Faculty of Engineering, Pontificia Universidad Católica de Chile, Macul 6904411, Chile

**Keywords:** electrospraying, betalains, hydroxypropyl methylcellulose, gelatin, core/shell structure

## Abstract

Betalains are bioactive compounds with remarkable functional and nutritional activities for health and food preservation and attractiveness. Nevertheless, they are highly sensitive to external factors, such as oxygen presence, light, and high temperatures. Therefore, the search for new structures, polymeric matrices, and efficient methods of encapsulation of these compounds is of great interest to increase their addition to food products. In this work, betalains were extracted from red beetroot. Betacyanin and betaxanthin contents were quantified. Subsequently, these compounds were successfully encapsulated into the core of coaxial electrosprayed capsules composed of hydroxypropyl methylcellulose (HPMC) and gelatin (G). The effect of incorporating the carbohydrate and the protein both in the core or shell structures was studied to elucidate the best composition for betalain protection. Morphological, optical, and structural properties were analyzed to understand the effect of the incorporation of the bioactive compounds in the morphology, color, and chemical interactions between components of resulting electrosprayed capsules. The results of the thermogravimetric and encapsulation efficiency analysis coincided that the incorporation of beetroot extract in G in the core and HPMC in the shell resulted in the structure with greater betalain protection. The effectiveness of the core/shell structure was confirmed for future food applications.

## 1. Introduction

Betalains are water-soluble nitrogenous pigments derived from betalamic acid that is extracted from beets. They are composed of betacyanins (red color) and betaxanthins (yellow color). These pigments can protect against age-related diseases and are commonly added to dairy products and meats [1,2]. They are relatively powerful colorants with the advantages of having three times more coloring strength than anthocyanins and are stable in the pH range of 3 to 7. Therefore, they are suitable for coloring acidic to neutral foods [2,3,4]. Nevertheless, the stability of betalains is highly influenced by exogenous factors, such as temperature, pH, and the presence of oxygen and light during processing or storage [5,6]. Thus, various techniques have been studied to increase their stability in order to increase their incorporation in food products, such as the addition of antioxidants, chelating agents, and encapsulation [7,8,9]. Among these techniques, encapsulation has shown good performance in the stabilization and conservation of betalains. In addition, in the case of betalains from beetroot that are characterized by an earthy flavor due to the presence of geosmin and pyrazine, the encapsulation presents the extra advantage to minimize this off-flavor [10]. Several polymeric matrices and encapsulation technologies have been used to encapsulate betalains and other bioactive compounds, such as spray drying, emulsification, and lyophilization [11,12,13]. However, these techniques have some disadvantages, such as the need for high temperatures, non-uniform conditions in the drying chamber, lack of control of the particle size, and the dispersion of betalains throughout the structure of the capsule. The selection of the proper technology and the polymeric matrix and its porosity are the main factors that can contribute to the reduction of degradation of the encapsulated betalains. Processes that require high temperatures or the action of mechanical forces can deteriorate these active compounds. In addition, the end application and resulting protection of the encapsulated compound will depend on the physical properties and nature of the polymeric matrices [9,10,11,12,13].

The electrohydrodynamic atomization process is an innovative dynamic process able to encapsulate substances at low temperatures by applying an electrical charge to a polymeric solution, which is a great alternative for thermosensitive compounds [14]. Specifically, the coaxial setup is characterized by the ability to form structures in which the active compound remains in the core of the capsule. This fact can allow a high efficiency of encapsulation of active agents by generating a core/shell geometry that ensures their protection and can delay the release of encapsulated substances [15,16,17,18]. Carbohydrate polymers have been extensively used to encapsulate active compounds through uniaxial and coaxial electrohydrodynamic atomization and other processes, and therefore, could be used to increase the stability of betalains [18,19,20,21]. The combination of carbohydrates has also provided different characteristics to the matrix and contributed to greater betalain protection [9,22,23]. Particularly, maltodextrin, guar gum, Arabic gum, and modified starch are some of the wall materials that have revealed interest and have been used in freeze-drying and spray-drying microencapsulations. As an example, research by Chronati et al. (2015) demonstrated freeze-dried encapsulated beetroot extract in maltodextrin (MD) and gum Arabic retained their structural integrity up to water activities of 0.66 and 0.82, respectively. Thus, the attention in the search for new edible carbohydrate polymers for encapsulating sensitive compounds as betalains has increased over last years, and hydroxypropyl methylcellulose (HPMC), a carbohydrate that has been scarcely applied in electrohydrodynamic processes, is proposed as a good candidate in this present study. HPMC is an FDA and EU-approved cellulose derivative food additive commonly used as a thickening and emulsifier component in the food industry [24,25]. Although research on the encapsulation of substances into HPMC-based electrospun capsules has been rare, Smeets et al. (2020) have recently developed coaxial capsules using HPMC in the core and the shell copolymer poly(methacrylic acid-co-methyl methacrylate) for the proper encapsulation of darunavir drug and its release in the small intestine [26]. Some works have used this carbohydrate to produce nanofibers containing drugs through the electrospinning process for buccal delivery systems [27,28]. Additionally, the studies conducted on the encapsulation of betalains from purple cactus pear by spray-drying indicated greater protection of betalains when the matrix is composed of a protein and a polysaccharide [29,30]. Robert et al. (2015) encapsulated cactus pear pulp rich in betalains in soy bean protein isolate with maltodextrin through spray-drying and evidenced the protein and polysaccharide blend improved betalain stability at 60 °C [29]. On the other hand, Castro-Muñoz et al. (2015) selected gelatin and maltodextrin as wall materials for spray-drying encapsulation of purple cactus pear juice [30]. Gelatin (G) is a widely used protein for encapsulation thanks to its surface activity with a positive or negative charge depending on the pH. It has high spinnability and good behavior when mixing with other encapsulating substances [31,32,33,34]. Active compounds have been successfully encapsulated in gelatin through electrodynamic atomization processes for biomedical, dental, and food packaging purposes [35,36,37,38]. However, the combination of HPMC and G in the development of coaxial electrospun capsules for encapsulation purposes has never been detailed before in the literature.

The present work describes the encapsulation of betalains extracted from red beetroot (*Beta vulgaris*) by means of coaxial electrodynamic atomization using G and HPMC as encapsulation matrixes. Both capsules containing the red beetroot extract (Bet) in the core structure of the core/shell of protein/polysaccharide (G-Bet/HPMC) and vice versa (HPMC-Bet/G) were developed. In addition, the uniaxial encapsulation of betalains in G and HPMC (G-Bet and HPMC-Bet, respectively), as controls, was also carried out.

Thus, based on the aforementioned background, the first hypothesis that arises from this study is that HPMC is a carbohydrate that can offer greater protection than MD due to its different chemical composition and lower water solubility. In addition, the design of the coaxial encapsulation structure should offer greater betalain protection than the structures obtained by uniaxial electrospinning. Given the lack of studies on core/shell structures for encapsulation with carbohydrate and protein, the third uncertainty that arises was the deduction of which polymer will offer greater protection in the core and shell structures of the encapsulating capsule. In order to answer these hypotheses and questions, the developed coaxial and uniaxial structures were structurally, morphologically, and thermally characterized and their encapsulation efficiency was determined.

## 2. Materials and Methods

### 2.1. Materials and Reagents

Hydroxypropyl methylcellulose F50 LV, HPMC, (code 35506), PM X11907 was supplied by Dimerco (Santiago, Chile) and gelatin, G, bloom 150° MSPPI00211, was purchased from Frutaron (Santiago, Chile). Folin Ciocalteu phenol reagent, anhydrous sodium carbonate (ACS reagent, anhydrous, ≥99.5%, powder), and gallic acid were purchased from Sigma Aldrich Química S.A (Santiago, Chile).

Beetroot (*Beta vulgaris*) was bought from a local supermarket (Santiago, Chile). Absolute ethanol for analysis (EMSURE^®^ ACS, ISO, Reag. Ph Eur) and acetic acid (glacial) 100%, anhydrous for analysis (EMSURE^®^ ACS, ISO, Reag. Ph Eur) was supplied by Merck (Santiago, Chile).

### 2.2. Preparation of Beetroot Extract

Beetroots were washed, peeled, and cut into cubes (2 cm × 2 cm), scalded in hot water at 70 °C for 2 min, and placed in an ice bath to quickly diminish the temperature.

Betalains (betacyanin and betaxanthin) were extracted under 50% (*v*/*v*) ethanolic solution with a 1:10 solid to solvent ratio. Scalded beetroot cubes were grounded using a hand blender Oster 2609 (Oster, China), further on the extraction was carried out at 40 °C under magnetic stirring at 150 rpm for 3 h. The mixture was filtered and centrifuged at 4000 rpm (Sigma 2-6E centrifuge, Osterode am Harz, Germany) for 10 min. The supernatant was collected and concentrated under vacuum using a rotator evaporator (Heidolph, Schwabach, Germany) at 40 °C until obtaining a final solution of 1 g mL^−1^ named “Bet”. The concentrated betalain extract was also lyophilized for further analysis and named as “BRE”. The resulting % solids were calculated as the percentage of solids present in the concentrated beetroot extract Bet.

#### Betalain Content Determination

Betacyanin (B_c_) and betaxanthin (B_x_) contents were determined by using UV–Visible spectrophotometer (6715 UV/Vis Jenway, Dunmow, England). Betalains content (B) was calculated following Equation (1) [39]:

B (mg per g) = (A·D_f_·M_w_·V_d_)/ϵ·L·W_d_ (1)

where A is the maximum absorption value at 535 and 483 nm for B_c_ and B_x_, respectively; D_f_ is the dilution factor; V_d_ is the volume of the pulp solution (mL), W_d_ is the weight of the pulp (g), and L is the path length (1 cm) of the bucket. For B_c_ quantification, the molecular weight (M_w_) is 550 g mol^−1^ and the molar extinction coefficient (ε) is ε= 60,000 L mol^−1^ cm^−1^ in water. In the case of B_x_, M_w_ is 308 g mol^−1^, and ε = 48,000 L mol^−1^ cm^−1^.

### 2.3. Microencapsulation of Beetroot Extract

**Figure 1:**
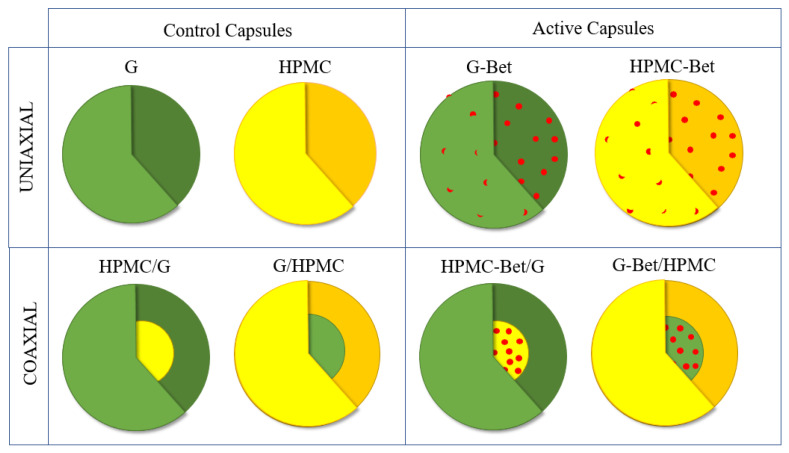
Scheme of developed electrosprayed capsules. In green: Gelatin (G); yellow: hydroxypropyl methylcellulose (HPMC); and red: beetroot extract (Bet). A lighter and darker color tone (green or yellow) was used to better represent the outer surface and inner bulk of the capsules, respectively. (Note: the colors of each component are for illustrative purposes only).

Active uniaxial and coaxial capsules containing betalains from beetroot extract (Bet) were obtained by electrodynamic atomization and their structures are schematically shown in Figure 1. Encapsulant polymers were HPMC and G, and the coaxial structures were obtained by combining both polymers into both inner (core) and outer (shell) structures. The control electrosprayed samples (capsules without beetroot extract) were also developed in order to study the effect of incorporating betalains in the electrosprayed systems. The resulting developed capsules were:

(a) Uniaxial structures: (1) HPMC, (2) HPMC-Bet, (3) G, and (4) G-Bet.
(b) Coaxial core/shell structures: (1) HPMC/G, (2) HPMC-Bet/G, (3) G/HPMC, and (4) G-Bet/HPMC.

An amount of 1 g of gelatin (G) was dissolved in 20 mL 40% aqueous acetic acid with constant stirring at 40 °C for 1 h. An amount of 0.8 g of HPMC was dissolved in 20 mL of ethanol 50%. Additionally, Bet was also incorporated to G and HPMC solutions at 14% wt. relative to the total mass content in the capsules. The developed uniaxial and coaxial capsules and their composition are reported in Table 1.

Electrospraying was carried out using electrospinning equipment (Spraybase^®^ power supply unit, Maynooth, Ireland) with a standard vertical configuration equipped with two pumps, a high-voltage power and a collector plate covered with aluminum foil connected to the grounded counter electrode of the power supply. HPMC/G, G/HPMC, HPMC-Bet/G, and G-Bet/HPMC core/shell capsules were electrosprayed by using a concentric stainless steel needle (0.7 mm/1.2 mm diameters) with a distance between the tip of the capillary and the collector plate of 12 cm, internal and external fluxes of 0.05 mL h^−1^ and 0.15 mL h^−1^, respectively, and a voltage between 14 and 16 kV.

The uniaxial control capsules of HPMC, G, hydroxypropyl methylcellulose containing betalains extract (HPMC-Bet), and gelatin containing betalains extract (G-Bet) were electrosprayed using a 0.9 mm diameter capillary with a distance between the tip of the capillary and the collecting plate of 12 cm, an injection flow of 0.15 mL h^−1^ and a voltage between 12 and 14 kV.

### 2.4. Characterization of the Electrosprayed Capsules

#### 2.4.1. Morphological Analysis

The morphologies of the control and active capsules were analyzed through scanning electron microscopy (SEM). The capsules were placed onto a strip of adhesive tape and then coated with gold (~5 nm) at a speed of 0.1 nm s^−1^ under an argon pressure of 0.05 mbar. The samples were examined using Quanta™ 250 FEG-SEM (Thermo Fisher Scientific, Waltham, MA, USA) at an accelerating potential of 20 kV, using 10 and 30 kx magnifications. The image analysis was performed with ImageJ program (Bethesda, MD, USA). Particle size was determined as the maximum caliper or Feret’s diameter, defined as the longest distance between any two points along the particle. Circularity was used to describe the shape of isolated particles, where C is equal to 1 for a circle, and less than 1 for less circular shapes, and it was calculated as follows:

Circularity = (4∙π∙area)/perimeter^2^ (2)

#### 2.4.2. Optical Properties

Lightness (L*) and chromaticity (a* and b*) parameters were measured in a CR-410 Minolta chroma-meter colorimeter (Minolta, Osaka, Japan) using the CIELab scale with a D65 illuminant and a 2° observer. In addition, to evaluate the color changes produced by the incorporation of Bet, the color differences (△E*) were calculated with respect to the G and HPMC control capsules following Equation (3) [40]:

△E* = [(△L*)^2^ + (△a*)^2^ + (△b*)^2^]^1⁄2^ (3)

where △E* is the color difference; △L*, △a* and △b* corresponded to the difference between the values of each parameter for G or HPMC capsules containing Bet and the G or HPMC control capsules. Color results were the average of three measurements.

#### 2.4.3. Structural Analysis

Attenuated total reflectance Fourier transform infrared spectroscopy (ATR-FTIR) was used to identify the specific functional chemical groups of the carbohydrate and protein capsules. FTIR spectra were performed through spectrometer equipment in ATR mode with a Bruker IFS 66V spectrometer (Ettlingen, Karlsruhe, Germany). The spectra were the result of 64 co-added interferograms at 2 cm^−1^ and resolutions in the wavenumber range from 4000 to 400 cm^−1^. The spectra analyses were performed using OPUS Software Version 7.

### 2.5. Encapsulation Efficiency (EE)

The encapsulation efficiency (EE) was determined following the procedure of Idham, Muhamad, and Sarmidi (2012) with some modifications [41,42] in order to evaluate the effectiveness of encapsulation. This test consisted of the determination of total polyphenols (TP) and surface polyphenols (SP) of the active capsules containing Bet. 0.01 g of each active capsule was mixed with 1 mL of distilled water in an Eppendorf tube to measure SP. 0.01 g of each active capsule was mixed with 1 mL of 50% ethanol solution acidified with 20% acetic acid for TP measurement. In both cases, the samples were vortexed for 30 s. Finally, each sample was filtered through a 0.22 µm filter and the supernatants were analyzed following the Folin–Ciocalteu method with slight modifications [43,44]. An amount of 100 µL of extract was mixed with 3100 µL of distilled water and 200 µL of Folin–Ciocalteu reagent. The mixture was shaken and stored in the dark for 5 min, and subsequently, 600 µL of anhydrous sodium carbonate (20% wt.) was added and stirred. After 2 h of reaction, the coloration of samples was measured at 765 nm. %EE was calculated following Equation (4):

%EE (%) = (TP−SP)·TP^−1^·100 (4)

The experiment was performed for all electrosprayed capsules containing betalains and repeated three times.

### 2.6. Thermal Stability Characterization

An amount of 2 to 3 mg of each sample (lyophilized beet extract and electrosprayed capsules) was heated from 30 °C to 600 °C with a heating rate of 10 °C min^−1^ under a nitrogen atmosphere in a Mettler Toledo Gas Controller GC2 Stare System TGA/DSC unit (Schwerzenbach, Switzerland). The onset decomposition temperature (T_onset_) and the temperature at the maximum degradation rate (T_d_) were recorded using the STARe software (Mettler Toledo, Schwerzenbach, Switzerland). Thermogravimetric analyses were carried out in duplicates.

### 2.7. Statistical Analysis

A random-type experimental design was used. Data analysis was carried out using Statgraphics Plus 5.1. This software was used to implement variance analysis and Fisher’s LSD test. Differences were considered significant at *p* < 0.05.

## 3. Results

### 3.1. Chemical Characterization of Beetroot Extract

The analysis of betalain content through the spectrophotometric method resulted in B_x_ and B_c_ values of 0.633 ± 0.003 and 0.663 ± 0.004 mg per 100 g of fresh beetroot, respectively. Thus, the total betalain content was 1.296 ± 0.005 mg per 100 g of fresh beetroot. The solid content of concentrated beetroot extract Bet was 13.54%.

### 3.2. Morphological and Optical Characteristics

**Figure 2:**
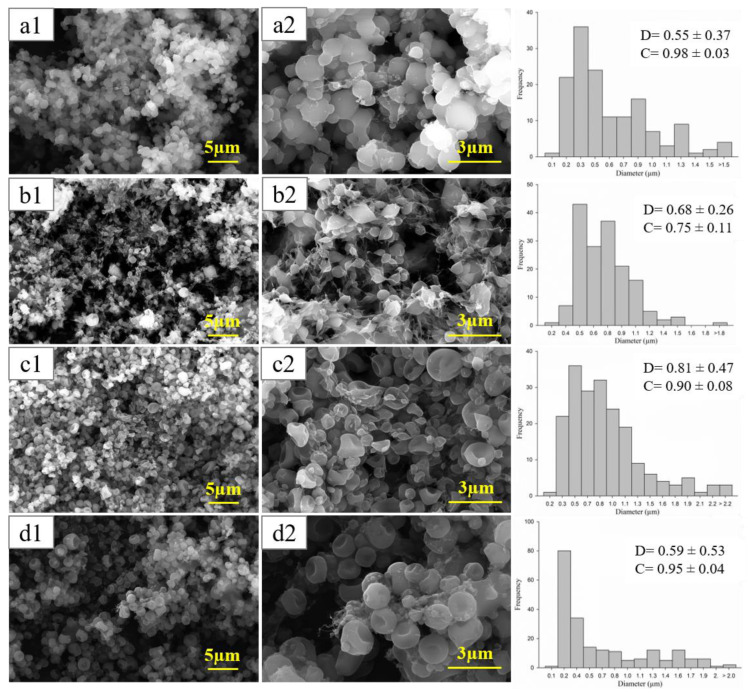
SEM micrographs of the capsules (**a**) G-Bet, (**b**)HPMC-Bet, (**c**) G-Bet/HPMC y (**d**) HPMC-Bet/G. SEM images at the left (**1**) and right (**2**) correspond to 10 kx and 30 kx magnifications, respectively. D is the average diameter (µm) and C circularity results.

Figure 2 shows SEM microscopies of coaxial and uniaxial capsules containing Bet at two magnifications and the histograms of their size distribution. In general, SEM micrographs of active electrosprayed capsules evidenced spherical shapes and some irregular and compressed capsules with smooth surfaces and variation in the particle sizes depending upon the encapsulating matrixes.

A surface of 255.5 µm^2^ was analyzed. Most of the capsules with sizes over 1 μm were collapsed (depressed center). In the case of G-Bet, a few isolated capsules that could be dimensionally characterized were observed. Several of these capsules were “melted”. Only small sized capsules maintained their shape although not those of larger sizes.

The coaxial structures presented some irregular shapes most likely produced by wall collapse. This was possibly due to the evaporation of the solvents with different volatility from the inner and outer structures during the electrodynamic atomization processes.

Regarding the circularity parameter, interestingly, capsules were more circular when they contained a higher content of G with respect to the total mass of the capsule, which is 0.75, 0.90, 0.95, and 0.98 for 0, 25, 68 and 86 wt% of G (Table 1). On the contrary, the circular shape was more affected when the HPMC content in the capsule increased (Figure 2b,c) possibly associated with its more rigid backbone.

Table 2 shows the color results of electrosprayed capsules. The uniaxial G and HPMC control capsules presented high luminosity values (L* = 92.10 and 92.55, respectively), a negative parameter a* near zero, and negative b* near −2 (slightly blue tones). Both HPMC/G and G/HPMC core/shell control capsules showed a decrease in luminosity values and slight changes in the a* and b* parameters, probably due to the presence of both layers with different compositions.

Electrosprayed capsules containing beetroot extract evidenced significantly higher color values compared to their control capsules. This finding indicated the incorporation of the pigment in the matrices. The uniaxial G-Bet capsule presented a large decrease in luminosity and the highest ΔE* value composed by the appearance of red (a* = 15.54) and yellow (b* = 22.02) tones. On the other hand, uniaxial HPMC-Bet capsules showed the lowest color difference (ΔE* = 13.72) with respect to the polymeric matrix, through the presence of lower red (a* = 3.86) and yellow (b* = 9.14) parameter values. The color parameters indicated slightly smaller differences than those recorded in G-Bet capsules. This fact was due to the higher encapsulation degree and protection afforded by the coaxial structure where the active components were incorporated in the core of the capsules.

In general terms, the incorporation of Bet in the uniaxial and coaxial capsules caused a decrease in luminosity and an increase in the a* parameter due to the appearance of red tones caused by the presence of B_c_, and an increase in the b* parameter due to the yellow tones provided by B_x_. All the active capsules presented yellow tones predominating over red ones (a* < b*), except for the HPMC-Bet/G core/shell capsules, whose red tones predominated over their yellow ones. Regarding the color difference of the active capsules, the perception of color followed this order: HPMC-Bet < G-Bet/HPMC < HPMC-Bet/G < G-Bet, concluding that the structures with a higher proportion of G showed more red and yellow tones compared to the structures with higher HPMC composition. Nevertheless, when compared with other studies of betalain encapsulations, resulting microcapsules have shown significantly higher values of corresponding color parameters [9,30]. Castro-Muñoz et al. (2025) developed microcapsules with values between 17.26–40.90 and 6.26–15.46 for a* and b* parameters, respectively [30].

### 3.3. Structural Properties

**Figure 3:**
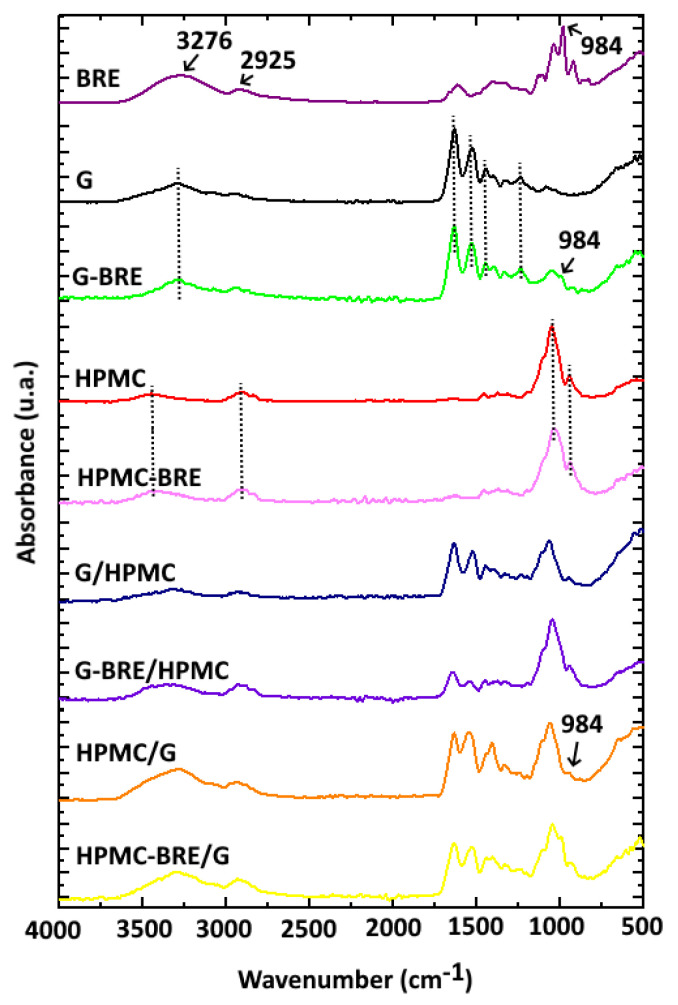
FTIR spectra of the uniaxial and coaxial core–shell capsules based on G, HPMC, and lyophilized beetroot extract, BRE.

The intermolecular interactions between the components were analyzed through ATR-FTIR. Figure 3 shows the FTIR spectra of uniaxial control capsules (G and HPMC) and the uniaxial capsules loaded with betalains (G-Bet and HPMC-Bet) in order to observe if the incorporation of Bet entailed the appearance of new peaks or the displacement of some bands.

Uniaxial G capsules presented the typical characteristic peaks of this protein matrix at 3278 cm^−1^ (stretching vibration of hydroxyl groups), 1632 cm^−1^ (stretching vibration carbonyl group of amide I), 1540 cm^−1^ (N-H vibration bending of amide II), and 1240 cm^−1^ (N-H stretching of amide III) [45,46]. Meanwhile, the uniaxial HPMC capsules presented the main bands at 3444 cm^−1^ (O-H vibrational stretching), 2900 cm^−1^ (methyl and hydroxyl propyl symmetric stretching), 1640 cm^−1^, 1338 cm^−1^, and 1045 cm^−1^ (stretching vibration of ethereal C-O-C groups) [47,48]. The position of the characteristic peaks of the uniaxial G and HPMC capsules containing betalains was maintained, confirming the low number of intermolecular interactions between these polymeric matrices and the compounds in the natural extract. This low interaction is probably due to their low concentration (Figure 3). The same behavior was reported for betalains from an amaranth extract and fish gelatin films [49]. Nevertheless, the physical presence of betalains in the uniaxial G capsules was verified through the appearance of a new peak at 984 cm^−1^ associated with its typical aromatic ring vibration [50]. Other characteristic peaks of betalains, such as bands at 3276 cm^−1^ (O–H stretching), 2925 cm^−1^ (C–H stretching), 1612 cm^−1^ (asymmetric C=C stretching), and 1401 cm^−1^ (C–C stretching), were probably overlapped with those of G and could not be observed due to the low betalain concentration in the samples [51,52]. Analogously, this could also explain the complete overlapping of HPCM peaks with characteristic betalains–beetroot extract bands in the uniaxial HPMC-Bet capsules.

Figure 3 also shows the FTIR spectra of the control and active G/HPMC and HPMC/G core/shell capsules. The characteristic peaks of G and HPMC were confirmed in the developed core/shell structures, but with less intensity due to their lower concentration regarding the uniaxial structures. This indicated no intermolecular interactions between the polymers, a fact that was previously reported for G and HPMC blended films [53]. Surprisingly, the presence of betalains was evidenced in the HPMC-Bet/G core/shell capsules through the presence of a peak at 988 cm^−1^, probably due to the low HPMC concentration (18 wt%) in this core/shell system. Meanwhile, this peak was completely overlapped by the intense HPMC bands when this polymer was used as a shell structure in the G-Bet/HPMC capsule due to its higher concentration (61 wt.%). The same behavior was observed in the uniaxial HPMC-Bet capsule.

### 3.4. Encapsulation Efficiency Results

**Figure 4:**
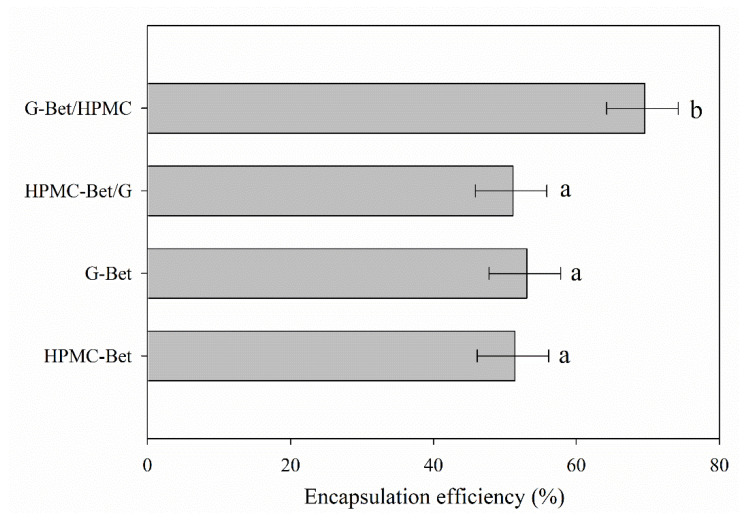
Encapsulation efficiency results of electrosprayed capsules (a and b denote statistically significant differences (*p* < 0.05) between EE of developed capsules).

The encapsulation efficiency (EE) value is normally defined as the precise number of active compounds that are actually protected in a system [54]. Figure 4 displays the encapsulation efficiency values obtained for each electrosprayed capsule. The G-Bet/HMPC capsule showed the highest %EE of approx. 69.3% probably thanks to the lower solubility in water of HPMC and greater affinity between gelatin and Bet that stabilized betalains in the core of coaxial capsules. The other electrosprayed capsules exhibited similar %EE results without significant differences. The highest %EE of betalains in the literature have been found when using lecithin in nanoliposomes, sodium alginate, and maltodextrin when applying techniques such as hydration–sonication, gelation, and spraying drying processes, respectively, achieving values of 80.35, 89.82, and 100%, respectively [54,55,56].

In general, %EE results were lower than expected because the polymers used probably manifested an important degree of dissolution during the determination of surface polyphenols. The methodology used to determine this parameter and the interaction with the encapsulating polymers could affect the data obtained. Although G and HPMC do not dissolve in distilled water, probably a large part of the polymers that were part of the coaxial and uniaxial capsules was slightly dissolved or dragged during the SP analysis, causing the release of betalains including betalains located inside the capsule (measured as polyphenols). Additionally, the solubility of the developed capsules was probably increased by their smaller size of approx. 550–800 nm.

Thus, although this parameter is interesting because it can be used to compare process efficiencies, the nature of the encapsulating polymers must be taken into consideration as to how the methodology can affect the results.

### 3.5. Thermal Stability Results

**Figure 5:**
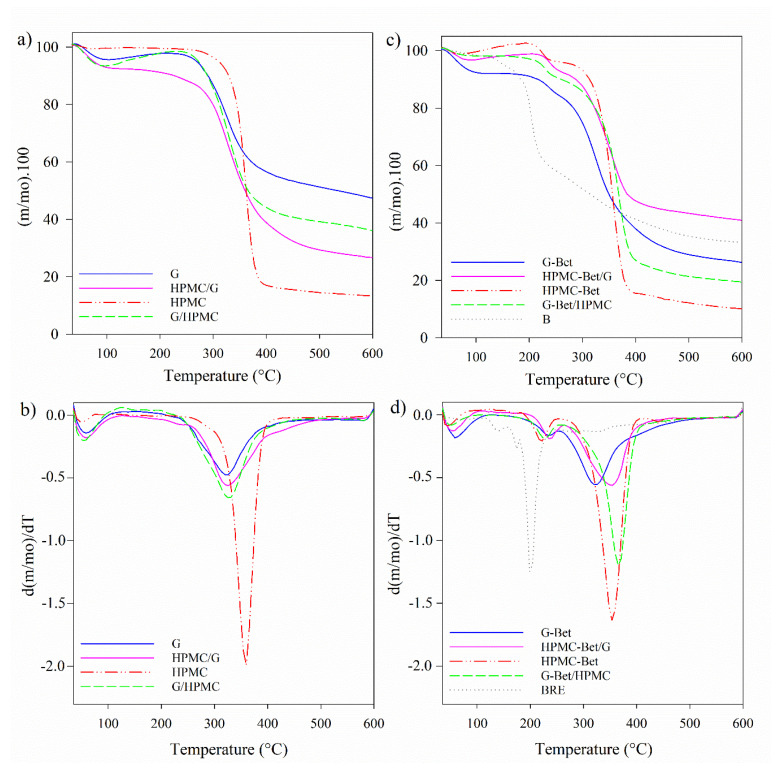
TGA and DTGA curves of (**a**,**b**) the control capsules, and (**c**,**d**) lyophilized beetroot extract (BRE) and active capsules. Solid and dash lines correspond to the capsule structures with higher concentrations of G and HPMC, respectively.

The degradation behavior of BRE and the control and active capsules based on G and/or HPMC are shown in TGA and DTGA curves in Figure 5 and Table 3. These measurements are an indirect analysis that can contribute to predicting the behavior of these protein and carbohydrate-based capsules when exposed to high temperatures, such as cooking, baking, or some preservation techniques.

All samples presented a mass loss at 120 °C associated with the evaporation of adsorbed water (Figure 5). As Table 3 shows, BRE initiated its mass loss at 127 °C (Figure 5c) and presented peaks with T_d_ at 144 °C and 204 °C, like those reported for the industrial dye E-162 from beetroot extract, which had a mass loss of 6.7% at 140 °C, and subsequently, BRE presented a significant fast degradation with a mass loss of 31.9% at 213 °C [57]. In addition, BRE degraded in several stages above 120 °C related to the decomposition processes of particle components such as proteins and carbohydrates. This could be associated with the decomposition of BRE components according to their molecular weight, as the low molecular weight components with high content of sugars are the most susceptible to degradation. Sucrose, fructose, glucose, and acids have low glass transition temperatures [22,58].

Regarding the uniaxial control capsules, gelatin capsules presented a degradation stage between 270 °C and 480 °C with the T_d_ at 326 °C, associated with the rupture of the helical structure of the protein chains and the rupture of peptide bonds, respectively (Table 3) [59]. Meanwhile, HPMC was more thermally stable, and the mass loss started at 338 °C with a maximum degradation rate of 361 °C due to the degradation of cellulose ethers, which included the dehydration and demethylation process [24,60].

On the other hand, the coaxial control capsules presented a single stage of mass loss between 250 °C and 500 °C attributed to the degradation of both polymers, with a maximum degradation rate at T_d_ of 330 °C approx. This temperature value was closest to the T_d_ of G. Meanwhile, the T_onset_ of the control coaxial capsules were between the T_d_ values of the uniaxial capsules for each polymer and the T_d_ of the capsules without Bet, following the order: G < HPMC/G < G/HPMC < HPMC, in accordance with the gelatin composition of the capsules: 100, 79, 29, and 0% wt., respectively. Although G/HPMC and HPMC/G evidenced similar T_d_, G/HPMC initiated its mass loss at lower temperatures.

With regard to the active capsules, TGA allowed analyzing the effect of the coaxial structure on the thermal protection of the beetroot extract. All active coaxial structures displayed two degradation stages that can be attributed to a first and second mass loss due to the degradation of betalains and the polymeric matrices, respectively (Table 3 and Figure 5d). The T_onset_ of the active capsules were lower than the T_onset_ of their corresponding control capsules due to the addition of beetroot extract which is more thermally unstable. However, it is noteworthy that in the active capsules: (i) T_onset_ and T_d,1(B)_ were significantly higher than in the pure BRE; (ii) a single degradation stage of the polymeric phase was observed in the HPMC capsules with T_d_ between 356 °C and 369 °C, temperatures closer to the T_d_ of HPMC; and (iii) T_d,1(Bet)_ increased in the following order: BRE < HPMC-Bet< G-Bet/HPMC< G-Bet< HPMC-Bet/G. Consequently, HPMC-Bet/G capsule showed the highest thermal degradation protection showing the highest T_onset_ (227 °C) and T_d_ (240 °C and 356 °C) (Table 3). The thermal protection of Bet in coaxial capsules can be attributed to the formation of a polymeric system with combined properties of the protein (G) and the polysaccharide (HPMC), being higher when the Bet was encapsulated in the more thermally stable polymer (polysaccharide) surrounded with the protein shell. Similarly, Robert et al. (2015) evinced that the encapsulation of cactus pear pulp by spray-drying with a mixture of soy protein isolate (SPI) and inulin (SPI + I) or maltodextrin (SPI + MD) improved betalain stability at 60 °C [29].

In addition, it can be noted that the beginning of the degradation stage of the polymers in the active capsules shifted to higher temperatures due to the antioxidant effect of Bet (Figure 5b,d), reaching temperatures of 283 °C, 314 °C, and 301 °C for G-Bet, G-Bet/HPMC, and HPMC-Bet/G, respectively, compared to the corresponding control capsules with 270 °C, 278 °C, and 287 °C, respectively (Table 3). Exceptionally, in the case of the HPMC-Bet capsule, the incorporation of Bet slightly anticipated HPMC degradation.

## 4. Conclusions

The encapsulation of a betalain-rich beetroot extract through an electrodynamic atomization process with a coaxial system using gelatin and hydroxypropyl methylcellulose as encapsulating polymers was successfully carried out, obtaining capsules with core/shell structures with the protein and the carbohydrate in both sections of the coaxial capsule. They presented irregular shapes from wall collapse produced by the evaporation of the solvents with different volatility from the inner and outer structures formed during electrodynamic atomization processes. The circularity values of electrsprayed capsules increased with the gelatin content. The resulting encapsulation efficiencies of the developed capsules were lower than expected, and the highest EE was obtained for the G-Bet/HPMC capsule, which also evidenced the highest temperature of decomposition of polymeric matrices.

## Figures and Tables

**Table 1:** Theorical composition of the electrosprayed capsules.

Capsules	HPMC (%)	G (%)	Bet (%)
G	0	100	0
HPMC	100	0	0
G/HPMC	71	29	0
HPMC/G	21	79	0
G-Bet	0	86	14
HPMC-Bet	86	0	14
G-Bet/HPMC	61	25	14
HPMC-Bet/G	18	68	14

**Table 2:** Color parameters of uniaxial and coaxial capsules.

Capsules	L*	a*	b*	ΔE*
G	92.10 ± 0.01 ^g^	−0.21 ± 0.02 ^bc^	−2.17 ± 0.01 ^b^	-
G-Bet	70.43 ± 0.02 ^a^	15.54 ± 0.04 ^f^	22.02 ± 0.03 ^e^	36.09 ± 0.0 ^d^
G/HPMC	88.56 ± 0.02 ^e^	−0.33 ± 0.01 ^b^	−2.64 ± 0.01 ^a^	-
G-Bet/HPMC	83.79 ± 0.19 ^c^	7.02 ± 0.34 ^e^	13.94 ± 0.87 ^d^	18.76 ± 0.68 ^b^
HPMC	92.55 ± 0.21 ^h^	−0.10 ± 0.01 ^c^	−1.92 ± 0.02 ^b^	-
HPMC-Bet	85.48 ± 0.09 ^d^	3.85 ± 0.16 ^d^	9.14 ± 0.65 ^c^	13.72 ± 0.59 ^a^
HPMC/G	90.56 ± 0.01 ^f^	−0.52 ± 0.01 ^a^	−2.75 ± 0.01 ^a^	-
HPMC-Bet/G	73.53 ± 0.02 ^b^	17.14 ± 0.03 ^g^	13.99 ± 0.03 ^d^	29.70 ± 0.01 ^c^

Each value represents the mean of 6 replicates with its respective standard deviation. The different superscript letters a–h denote statistically significant differences (*p* < 0.05) between capsules of the same color parameter.

**Table 3:** TGA parameters of lyophilized beetroot extract (BRE), control, and active capsules.

Sample	T_onset_ (°C)	T_d,1(Bet)_ (°C)	T_d,2(Pol)_ (°C)
BRE	127	144/204	-
G	270	-	326
HPMC	338	-	361
G/HPMC	278	-	330
HPMC/G	287	-	328
G-Bet	214	237	325
HPMC-Bet	211	224	358
G-Bet/HPMC	216	229	369
HPMC-Bet/G	227	240	356

BRE: Lyophilized beetroot extract. Pol: polymer. Active capsules with uniaxial (polymer-Bet) and coaxial (polymeric core Bet/polymeric shell) structures.

## Data Availability

Not applicable.
